# Characterization of the complete chloroplast genome of *Oxytropis aciphylla* Ledeb. (Leguminosae)

**DOI:** 10.1080/23802359.2022.2124822

**Published:** 2022-09-27

**Authors:** Zhanlin Bei, Lei Zhang, Xingjun Tian

**Affiliations:** a School of Life Sciences, Nanjing University, Nanjing, People’s Republic of China; b School of Biological Science and Engineering, North Minzu University, Yinchuan, People’s Republic of China

**Keywords:** *Oxytropis aciphylla* Ledeb., *Leguminosae*, complete chloroplast genome

## Abstract

To better understand the taxonomy of the genus *Oxytropis*, we sequenced the complete chloroplast genome of *Oxytropis aciphylla* Ledeb. The total plastome of *O. aciphylla* Ledeb. is 122,121 bp in length with a GC content of 34.3%. It contains one large single-copy (LSC) region of 88,235 bp, one small single-copy (SSC) region of 10,400 bp, and one inverted repeat (IR) region of 23,486 bp, encoding 76 proteins, four rRNAs, and 29 tRNAs. The phylogenetic position shows that *O. aciphylla* Ledeb. is the closest to *Oxytropis glabra*.

*Oxytropis* is one of the largest genera of the *Leguminosae* family (Malyshev 2008). It includes approximately 450 species and distributed in Inner Mongolia, Shaanxi, Ningxia, Gansu, Qinghai, Xinjiang, and other provinces of China, as well as in Western Siberia of Russia and southern Mongolia. It has been reported that Central Asia and West Asia are the most important centers for the speciation of *Oxytropis* (Kholina et al. 2021). However, due to the high diversity of *Oxytropis*, the taxonomy of this genus is poorly understood. The complete chloroplast genome barcode not only plays an important role in the identification of plant species, but also plays a crucial role in the taxonomic relationship of poorly studied species (Techen et al. 2014; Tekpinar et al. 2016). However, the current research on the entire chloroplast genome of *Oxytropis* species is still poor.

*Oxytropis aciphylla* Ledeb. is a dwarf cushion-shaped *leguminous* perennial subshrub in the genus *Oxytropis* (Ledebour 1831). It mainly grows on hill slopes and sandy Gobi deserts (Zheng et al. 2013). As a desert plant, it is popular for cattle and sheep in the early spring and is the major plant litter of sandy Gobi deserts (Li et al. 2015). The whole chloroplast genome information of *O. aciphylla* Ledeb. has not been reported in the NCBI database. Therefore, in this study, we sequenced and structurally characterized its chloroplast genes, aiming to provide a useful resource for future research on *Oxytropis* genetic evolution.

Fresh leaves of *O. aciphylla* Ledeb. were collected from the Gobi (38°08′53.92″ N,105°54′29.08″ E) of Helan Mountain in Yinchuan City, Ningxia Hui Autonomous Region. Total genomic DNAs were extracted from the fresh leaves using the CTAB method (Doyle and Doyle 1987). A library with an average length of 150 bp was constructed using the Nextera XT DNA library preparation reagents (Illumina, San Diego, CA) and sequenced by the Illumina NovaSeq 6000 platform. A total of 6.01 Gb of sequence reads were generated and edited using NGS QC Toolkit v2.3.3. Contigs were obtained from high-quality reads using the de novo assembler SPAdes 3.11.0 software (Bankevich et al. 2012) and annotated using plan software (Huang and Cronk 2015) with *Oxytropis glabra* as the assembly and annotation reference genome. The complete sequence was submitted to GenBank (OK143433), and the sample was stored at the Laboratory of Ecosystem, North Minzu University, Yinchuan (voucher specimen: NMU00047) (Zhanglei, email: zhangsanshi-0319@163.com).

The complete chloroplast genome of *O. aciphylla* Ledeb. is 122,121 bp in length with an overall GC content of 34.3%. It contains an 88,235 bp large single-copy (LSC) region, a 10,400 bp small single-copy (SSC) region, and a 23,486 bp inverted repeat (IR) region and encodes 109 genes, including 76 PCGs, four rRNA, and 29 tRNA. To confirm the phylogenetic position of *O. aciphylla* Ledeb. and understand its relationship with other species in Leguminosae, the complete chloroplast genomes of seven species in the Leguminosae family, including *Oxytropis glabra* (NC056975.1) (Liu et al. 2021), were collected and aligned with *O. aciphylla* Ledeb. Subsequently, a phylogenetic tree was constructed by IQTREE v1.6 with 1000 bootstraps replicates using the Best-fit model (Nguyen et al. 2015; Hoang et al. 2018). A final ML tree was constructed using *Caragana jubata* as the outgroup. As shown in Figure 1, the entire tree was separated into two major clades: *Oxytropis*, and *Caragana*, with one *Caragana* species branch, seven *Oxytropis* species branches. *O. aciphylla* Ledeb. has the closest relationship with *Oxytropis glabra*. We believe that our results will be highly beneficial to studies on species identification, phylogenetic relationships, and population genetics of *Oxytropis* species as well as evolution in the *Leguminous* family.

**Figure 1.**
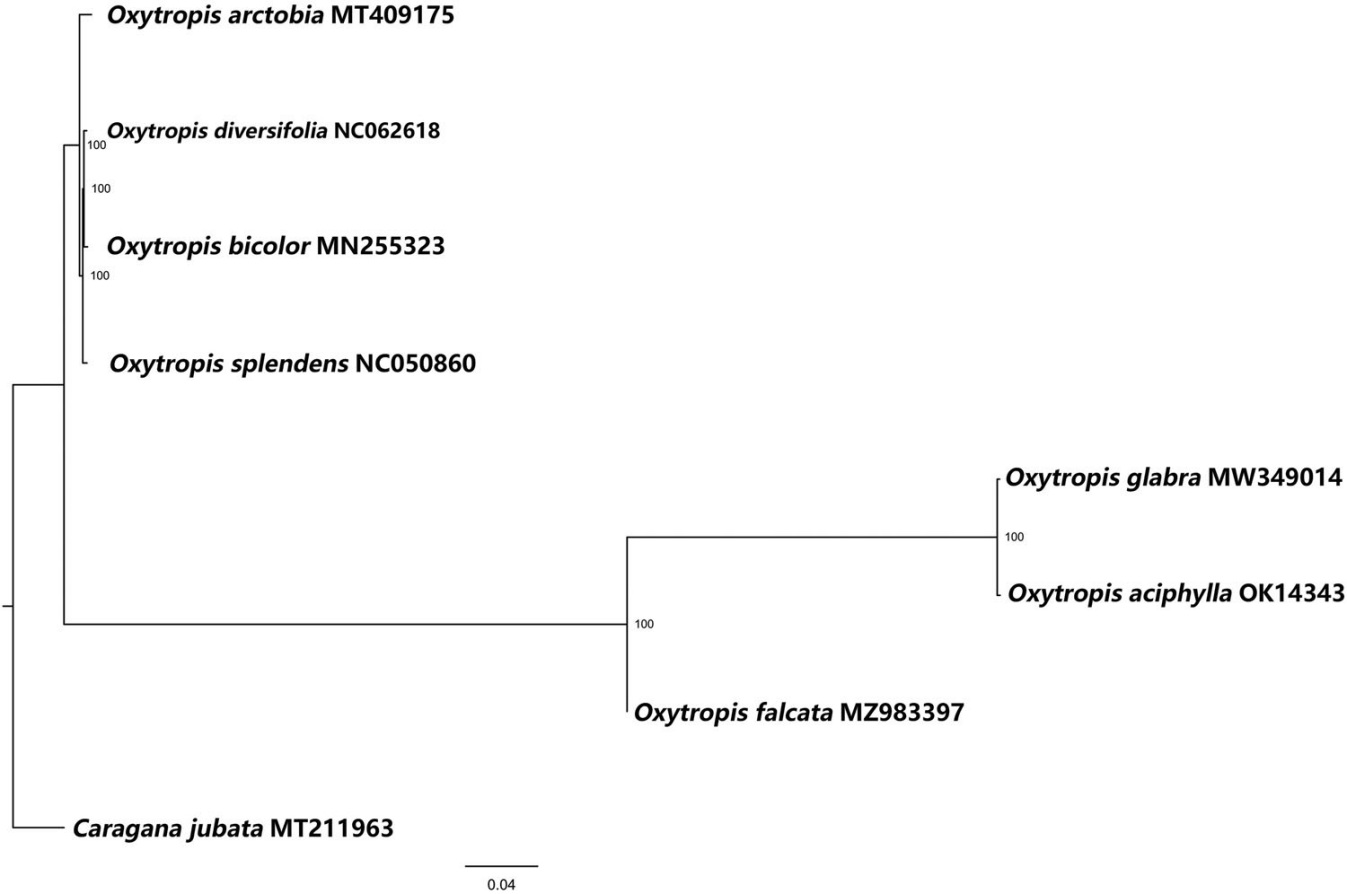
Maximum-likelihood phylogenetic tree for *Oxytropis aciphylla* Ledeb. based on complete chloroplast genomes of seven species in family *Leguminosae*, with *Caragana jubata* as the outgroup. The numbers to the right of the branches are bootstrap support values. At the bottom of the figure is the distance scale of phylogenetic tree.

## Data Availability

The chloroplast genome sequence data that support the findings of this study are openly available in GenBank of NCBI (https://www.ncbi.nlm.nih.gov/) under the accession number OK143433. The associated BioProject, SRA, and Bio-Sample numbers are PRJNA769668, SRR16249213, and SAMN22161435, respectively.
